# Treatment Effects of the Second-Generation Tyrosine Kinase Inhibitor Dasatinib on Autoimmune Arthritis

**DOI:** 10.3389/fimmu.2018.03133

**Published:** 2019-01-10

**Authors:** Kai Guo, Xin Bu, Chongfei Yang, Xiaorui Cao, Huan Bian, Qingsheng Zhu, Jinyu Zhu, Dawei Zhang

**Affiliations:** ^1^Department of Orthopaedics, Xijing Hospital, Fourth Military Medical University, Xi'an, China; ^2^State Key Laboratory of Cancer Biology, Department of Biochemistry and Molecular Biology, Fourth Military Medical University, Xi'an, China; ^3^Department of Orthopaedics, Shenzhen University General Hospital, Shenzhen University Clinical Medical Academy, Shenzhen, China

**Keywords:** rheumatoid arthritis, dasatinib, fibroblast-like synoviocyte, collagen-induced arthritis, tyrosine kinase

## Abstract

Rheumatoid arthritis (RA) is a multifactorial autoimmune disease that primarily manifests as persistent synovitis and progressive joint destruction. Imatinib exhibited a therapeutic effect in murine collagen-induced arthritis (CIA) via selective inhibition tyrosine kinases. The second-generation tyrosine kinase inhibitor dasatinib exhibits more durable hematological and cytogenetic effects and more potency compared to imatinib. However, the effect of dasatinib on CIA is poorly understood. The present study investigated the treatment effect of dasatinib on autoimmune arthritis. We demonstrated that dasatinib alleviated arthritis symptoms and histopathological destruction in CIA mice. Dasatinib treatment inhibited the production of proinflammatory cytokines including IL-1β, TNF-α, and IL-6, and promoted the production of the anti-inflammatory cytokine IL-10. Dasatinib treatment also suppressed the expression of anti-mouse CII antibodies including total IgG, IgG1, IgG2, and IgG2b, in CIA mice. We further demonstrated that dasatinib inhibited the migration and proliferation of fibroblast-like synoviocytes (FLS) from RA patients and promoted FLS apoptosis. The mRNA expression of MMP13, VEGF, FGF, and DKK1 was down-regulated in FLS treated with dasatinib. Our findings suggest that dasatinib exhibited treatment effects on CIA mice and that FLS are an important target cell of dasatinib treatment in autoimmune arthritis.

## Introduction

Rheumatoid arthritis (RA) is a chronic, systemic, autoimmune inflammatory disease that causes chronic inflammation in joints and subsequent cartilage destruction and bone loss. RA is a common disease that leads to musculoskeletal disability. Small joints such as those in the hands and feet are always involved in RA, and larger joints such as the knees are also frequently affected. RA affects ~0.5–1% of the adult population worldwide (1).

The mechanism of RA is not fully understood, but it is clear that fibroblast-like synoviocytes (FLS) play an important role in RA (2, 3). FLS contribute to inflammation and joint destruction. The synovium is a quiescent relatively acellular structure in normal people. Many factors such as high levels of cytokines, growth factors, and infiltrating inflammatory cells, stimulate the synovium in RA, and it becomes hyperplastic and invasive (4, 5). FLS migration and mobility increase and FLS apoptosis is restrained in RA patients. The synovium extends into the joint space and degrades the cartilage matrix. Synovial cells in RA patients invade and destroy cartilage and bone in joints. FLS strongly respond to the inflammatory environment and contribute to joint damage. The targeting of FLS may be an effective strategy for RA treatment.

Dasatinib is a second generation tyrosine kinase inhibitor that is used for the treatment of chronic myeloid leukemia or Philadelphia chromosome-positive acute lymphoblastic leukemia (6, 7). Dasatinib exhibits more durable hematological and cytogenetic effects and greater potency than the first-generation tyrosine kinase inhibitor imatinib (8). Dasatinib is a multitargeted inhibitor that inhibits BCR/ABL c-KIT, PDGFR, and the SRC family kinases such as SRC, LCK, HCK, YES, and FYN (9). Dasatinib also exhibits a better inhibitory effect on the collagen receptor tyrosine kinases discoidin domain receptor 1 (DDR 1) and discoidin domain receptor 2 (DDR 2) than imatinib and nilotinib (10). These tyrosine kinases play an important role in the pathological process of RA. Inhibition of these tyrosine kinases may be an effective treatment for RA. Previous studies demonstrated that imatinib and nilotinib exhibited therapeutical effects in mice with collagen-induced arthritis (CIA) (11, 12). Dasatinib shares similar targets with imatinib and nilotinib, but dasatinib is more potent than imatinib and nilotinib at inhibiting tyrosine kinases (13). Dasatinib exhibits its own specific targets, such as PI3K and ERK (14), which are potent targets in RA treatment (15, 16). However, the effect of dasatinib on CIA is not known. The present study evaluated the therapeutic potency of dasatinib in CIA mice and the effect of dasatinib on FLS from RA patients was further demonstrated.

## Materials and Methods

### Animals

Wild-type male DBA/1 mice (8–10 weeks old) were purchased from Cavens lab animal company (Changzhou, China). DBA/1 mice were singly housed at room temperature (22 ± 1°C) under a 12 h:12 h light-dark cycle with free access to water and food. The Ethics Committee of Xijing Hospital, Fourth Military Medical University, approved all animal and human experiments, which were performed in accordance with institutional regulations.

### Dasatinib

Dasatinib was obtained from Bristol-Myers Squibb (USA). Dasatinib solution was prepared as described previously. Dasatinib was dissolved in 80 mM citric acid (pH = 2.1) as a stock solution for *in vivo* experiments. The stock solution was diluted with citrate buffer (pH = 3.1) prior to therapeutic treatment. Dasatinib was dissolved in dimethyl sulfoxide (DMSO) as stock solution for cell experiments.

### Induction and Evaluation of Collagen-Induced Arthritis (CIA)

CIA was induced in 8–10 weeks-old male DBA/1 mice as previously described with some modifications. Chicken type II collagen (CII, Sigma, USA) was dissolved in 0.01 M acetic acid overnight at 4°C. Each mouse was injected intradermally at the base of the tail with 150 μg of type II collagen emulsified with an equal volume of complete Freund's adjuvant (CFA, Sigma, USA). Each mouse was boosted with an equal amount of 150 μg chicken type II collagen and an equal amount incomplete Freund's adjuvant (IFA, Sigma, USA) at the base of the tail 21 days after the first injection. Therapeutic treatment was initiated after the CIA model was established (arthritis score>2, approximately at day 28). CIA mice were randomly divided into a dasatinib group and vehicle group. Mice in the dasatinib group received gavage of 10 mg/kg dasatinib (approximately 0.2 ml) once daily from the 26th day to the 57th day. Mice in the vehicle group received gavage at the same time with the same volume of citrate buffer (pH 3.1). Mice were evaluated macroscopically for arthritis and the thickness of each paw was measured using microcalipers every 3 days. Mice were scored for clinical signs of arthritis in each paw on a scale of 0–4, where 0 = normal, 1 = swelling and/or redness in one joint, 2 = swelling and/or redness in more than one joint, 3 = swelling and/or redness in the entire paw, and 4 = maximal swelling.

### Micro CT Scan

Mice were euthanized via an intraperitoneal injection of pentobarbital sodium on the 58th day. Ankle and knee joint tissues were collected and scanned using an eXplore Locus SP Pre-Clinical Specimen micro-CT (GE Healthcare, USA) with a 14 μm resolution, 80 kV tube voltage and 80 mA tube current. The reconstruction and 3D quantitative analyses were performed using the software provided with the micro-CT system (GE Healthcare, USA). Bone trabecular in the distal femur with a thickness of 1 mm was defined as a region of interest (ROI). BMD, BV/TV, BS/BV, Tb.Th, Tb.N, and Tb.Sp were measured and analyzed in the ROI using the software of the micro-CT system for quantitative assessment (17, 18). The same settings for scans and analyses were used for all samples.

### Histopathological Examination

Mouse ankle joint tissues were fixed in 4% paraformaldehyde for 2 days and decalcified in 17% EDTA. Decalcified tissues were embedded in paraffin and sectioned. Sections (5 μm) were stained with hematoxylin and eosin to evaluate the morphology, and toluidine blue and safranin O were used to assess proteoglycans. Immunohistochemistry was performed using CD31. Sections were incubated with a CD31 primary antibody overnight at 4°C and incubated with a secondary antibody for 1 h. Sections were counterstained with hematoxylin. Sections were evaluated for bone and/or cartilage erosion, synovitis, and pannus formation based on a previously described scoring system: grade 0, normal; grade 1, mild inflammation, mild cell infiltration, mild hyperplasia of the synovial lining layer, mild cartilage destruction without bone erosion; and grades 2–4, increasing degrees of inflammatory cell infiltrates, synovial lining hyperplasia, and pannus formation and cartilage and bone destruction (19).

### Measurement of Serum Cytokine Levels

Peripheral blood was collected on day 58 followed by centrifugation at 2,000 rpm for 10 min. The levels of the inflammatory cytokines, including IL-1β, TNF-α, IL-6, and IL-10 were measured using ELISA kits (PeproTech, USA) in serum samples following the manufacturer's instructions. Serum levels of anti-mouse CII antibodies including total IgG, IgG1, IgG2, and IgG2b were measured using ELISA kits(Invitrogen, USA) according to the manufacturer's instructions.

### Cell Culture

Synovium tissues were obtained from RA patients undergoing total knee replacement in the Department of Orthopedics, Xijing Hospital. Two experienced doctors diagnosed RA patients in conformance to the American College of Rheumatology 1987 revised criteria. Synovium tissues were cut into small pieces and washed in cold sterile phosphate buffered saline (PBS). Then the small pieces of synovium tissues were digested in a serum-free DMEM solution with 1 mg/ml collagenase type I (Sigma, USA) in 5% CO_2_ and 37°C for 6 h with gentle rotation. Tissues were washed three times and resuspended in DMEM solution with 20% fetal bovine serum (FBS). Cells were cultured for 24 h and non-adherent cells were discarded. Adherent cells were cultured in DMEM solution with 20% FBS, 100 U/ml penicillin and 100 U/ml streptomycin in 5% CO_2_ and 37°C. Cells from passages 3–5 were used in the experiments.

### Erasion Trace Test

The erasion trace test was used to assess the migration of FLS. Cells were seeded on 6-well plates. Cells were cultured to ~80% confluency, and parallel lines with 0.5 cm spacing were drawn using 200 μl sterile tips on the bottom of each well. The scraped-off cells were washed out. Cells were cultured in a 20% FBS-DMEM solution with or without 2.5 μg/ml dasatinib for 24 h. Images were obtained under a microscope at 0, 12, and 24 h. Migration was quantified via counting the number of cells that crossed the parallel line.

### Transwell Migration Assay

The transwell migration assay was also used to assess FLS migration. A total of 1 × 10^5^ cells were seeded into the upper chamber of a transwell insert (Corning, USA) with 8 μm porosity polycarbonate filters in 200 μl of serum-free DMEM with or without 2.5 μg/ml dasatinib. A 20% FBS-DMEM solution (600 μm) with or without 2.5 μg/ml dasatinib was added to the lower chamber. Cells in the upper chamber were gently removed after 24 h using cotton swabs. Cells that immigrated to the lower surface of the inserts were fixed with 95% ethanol and stained using crystal violet. Images were obtained under a microscope, and migration was quantified via counting the number of cells in each field.

### Cell Proliferation Assay

Cell proliferation ability was assessed using 5-bromodeoxyuridine (BrdU). Cells were seeded in 3 replicates in 48-well plates with a 6 mm-diameter cover glass in each well. Cells were cultured to approximately 70% confluency and treated with or without 2.5 μg/ml dasatinib for 24 h. Cells were incubated with 10 μM BrdU (Sigma, USA) for 60 min and fixed in 4% paraformaldehyde for 15 min. Fixed cells were permeabilized with 0.1% Triton X-100 and incubated with 2 N HCl for 30 min at 37°C and 0.1 M borate buffer for 10 min at room temperature. Cells were blocked with goat serum and incubated with an anti-BrdU antibody (BD Biosciences, USA, 1:100) at 4°C overnight. Cells were reacted with TRITC-labeled anti-rat IgG. The fluorescent signal was captured using confocal laser-scanning microscopy (Nikon, Japan).

### Cell Apoptosis Assay

Cell apoptosis was detected using flow cytometry and an Annexin V-FITC apoptosis detection kit (BD Biosciences, USA) according to the manufacturer's instructions. Cells were seeded in 3 replicates in 6-well plates and treated with or without 2.5 μg/ml dasatinib for 1, 3, and 5 d. Cells were harvested using trypsin-EDTA, washed twice with cold PBS, centrifuged and double-stained with annexin V-FITC and PI in binding buffer for 15 min in the dark at room temperature Stained cells were subjected to flow cytometry.

### Reverse Transcription Polymerase Chain Reaction

Total cellular RNA was extracted using RNAiso Plus (Takara, Japan) according to the manufacturer's instructions. Total RNA (500 ng) was reverse transcribed to cDNA using the PrimeScript™ RT reagent kit (Takara, Japan). RT-PCR was performed using the CFX96 (BIO-RAD, USA) instrument (BIO-RAD, USA), and individual PCRs were performed in 96-well optical reaction plates using SYBR® Premix Ex Taq™ (Takara, Japan) according to the manufacturer's instructions. Target gene (MMP13, DKK1, VEGF and FGF) expression was normalized to the reference human gene ß-actin. The 2-ΔΔCt method was used to calculate the relative gene expression. PCR was performed using a program of 40 cycles at 95°C for 5 s and 60°C for 30 s. PCR products were subjected to a melting curve analysis and a standard curve to confirm the correct amplification. All PCRs were performed in triplicate. The sense primer for β-actin was TGACGGGGTCACCCACACTGTGCCCATCTA, and the antisense primer was CTAGAAGCATTTGCGGTGGACGATGGAGGG. The sense primer for MMP13 was TCCTGGGCCAAATTATGGAG, and the antisense primer was TTGCCGGTGTAGGTGTAGATAGGAA. The sense primer for VEGF was GCAGAATCATCACGAAGTGG, and the antisense primer was GCATGGTGATGTTGGACTCC. The sense primer for FGF was GAAGAGCGACCCTCACATCAAG, and the antisense primer was CTGCCCAGTTCGTTTCAGTG. The sense primer for DKK1 was AGCACCTTGGATGGGTATTC, and the antisense primer was CACACTTGACCTTCTTTCAGGA.

### Statistical Analysis

All data are presented as the mean ± SEM and were analyzed using the Statistical Package for the Social Sciences (SPSS) 13.0 for Windows (Chicago, IL, USA). Groups were compared using independent-samples *t*-tests. *P* < 0.05 was considered significant.

## Results

### Dasatinib Treatment Attenuated RA Severity in CIA Mice

We first investigated the treatment effect of dasatinib in mice with CIA. The CIA model was established in DBA/1J mouse. CIA was induced via the injection of chicken CII emulsified in CFA intradermally at the base of the tail. DBA/1 mice were boosted with an injection of chicken CII emulsified in IFA on the 21st day. Mice in the dasatinib group received 10 mg/kg (20, 21) dasatinib from the day 26 to 57 orally once a day. Mice in the vehicle group received the same volume of citrate buffer (pH 3.1) at the same times.

**Figure 1:**
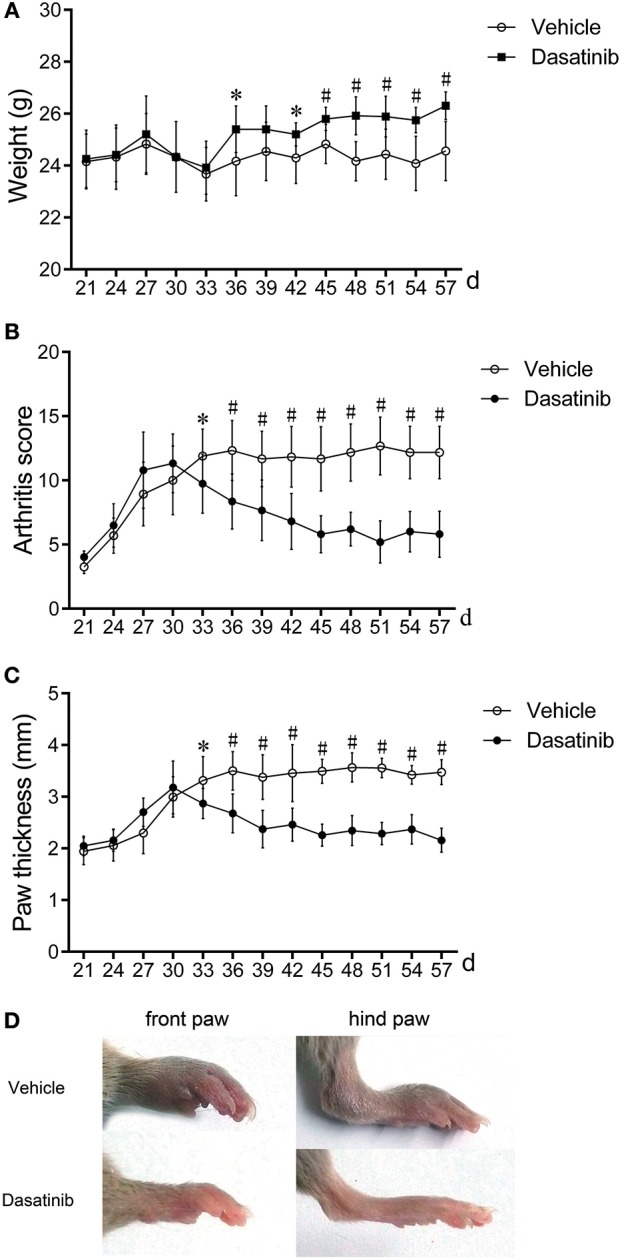
Treatment with dasatinib attenuated the severity of arthritis in mice with CIA. DBA/1J mice were immunized twice with chicken CII150 μg chicken type II collagen. Mice received gavage once daily from the 28th day to the 57th day with 10 mg/kg dasatinib and the same volume of control vehicle. All data are presented as the mean ± SEM. ^*^P < 0.05, #*P* < 0.01 vs. vehicle group (*n* = 9 per group). **(A–C)** Weight **(A)**, arthritis score **(B)**, and mean paw thickness **(C)** were calculated every 3 days from the 28th day to the 58th day after the first injection. **(D)** Macroscopic observations of the front and hind paws of mice on the 58th day.

The severity of CIA was evaluated using macroscopic observation, arthritis scores and paw thickness. The body weights of mice in the dasatinib group were markedly higher than the vehicle group (Figure 1A). Dasatinib treatment significantly decreased arthritis scores and paw thickness (Figures 1B,C). CIA developed as paw swelling and redness 26 days after the first injection. The plateaus of the peak arthritis scores and paw thickness were obtained after the 36th day. Macroscopic observation revealed significantly reduced paw redness and swelling in the dasatinib group compared to the vehicle group (Figure 1D).

### Dasatinib Treatment Alleviated Bone Destruction in Mice With CIA

**Figure 2:**
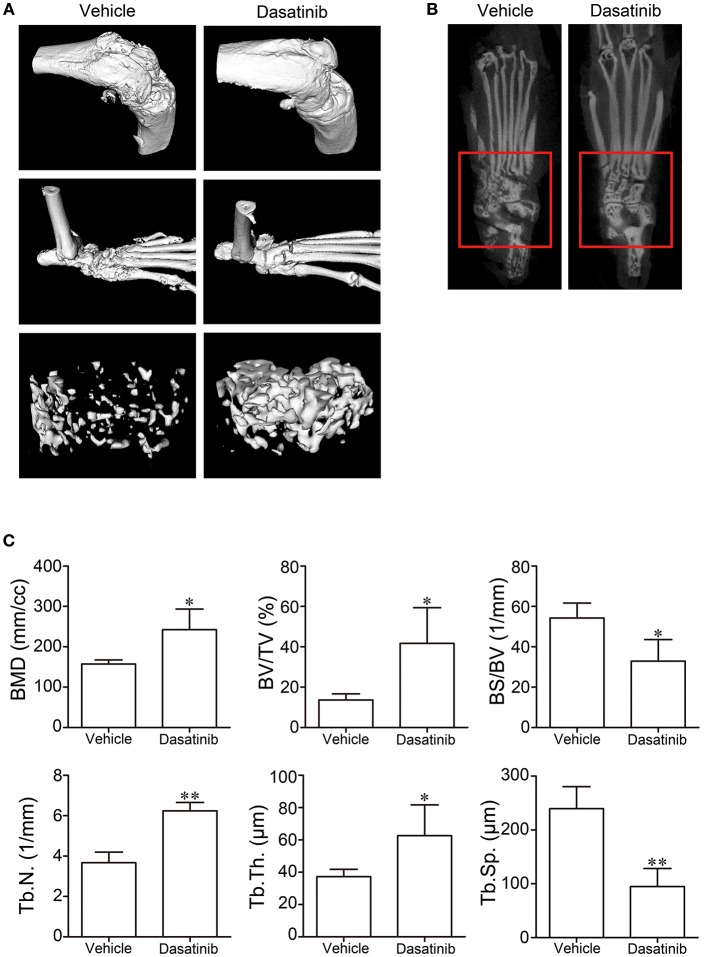
Micro-CT was used to evaluate bone destruction in CIA mice. **(A)** 3D model of knee joints, ankle joints, and bone trabecular in the distal femur reconstructed using the software of the micro-CT system. **(B)** 2D CT tomographic images of ankle joints. **(C)** 3D parameters of bone trabecular in distal femur analyzed using the software of the micro-CT system. ^*^P < 0.05, ^**^P < 0.01 vs. vehicle group (*n* = 4 per group).

Bone destruction of CIA mice was evaluated using micro-CT. The knee joints, ankle joints and bone trabecular in the distal femur were reconstructed using the software of the micro-CT system. Bone destruction was observed. The vehicle group exhibited severe bone erosion and large bone volume loss in the 3D model (Figure 2A) of reconstruction and 2D CT tomographic images (Figure 2B). The bone surface was crude and the joint space was unclear in the vehicle group. Bone erosion and the volume of bone loss were alleviated in mice in the dasatinib group. The bone surface was much smoother and the joint space was much clearer in ankle joints of CIA mice treated with dasatinib (Figures 2A,B). The following 3D parameters were used to analyze the bone trabecular and quantify the bone destruction level: bone mineral density (BMD), bone volume to tissue volume (BV/TV), bone surface to bone volume (BS/BV), trabecular thickness (Tb.Th), trabecular number (Tb.N), and trabecular separation (Tb.Sp). BMD, BV/TV, Tb.Th, and Tb.N were significantly higher in the dasatinib-treated group than the vehicle group, and BS/BV and Tb.Sp were markedly lower in the dasatinib-treated group than the vehicle group (Figure 2C). The quantified indexes demonstrated that dasatinib alleviated bone destruction in mice with CIA.

### Dasatinib Treatment Improved the Tissue Structure of the Ankle Joint in Mice With CIA

**Figure 3:**
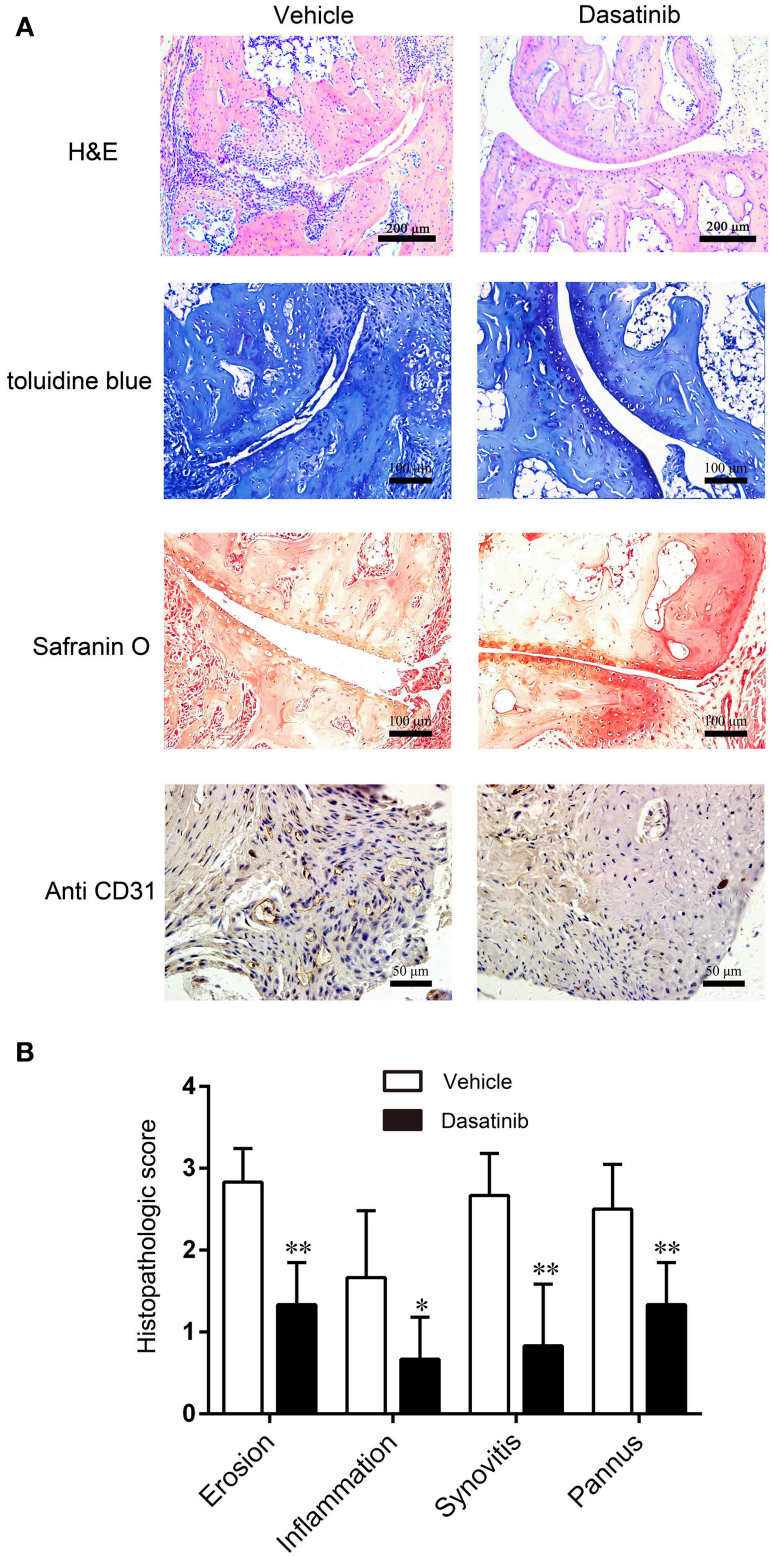
Histopathological analyses were performed on hind ankle joints to evaluate the tissue structure alterations in CIA mice. **(A)** H&E stain, toluidine blue stain, safranin O stain and CD31 immunohistochemistry stain of ankle slices. **(B)** Histology scores of synovitis, inflammation, cartilage damage, bone erosion, and pannus formation in ankle joints. ^*^P < 0.05, ^**^P < 0.01 vs. vehicle group (*n* = 6 per group).

Histopathological analyses were performed on hind ankle joints to evaluate the tissue structure alterations in CIA mice. Representative images of H&E stain, toluidine blue stain, safranin O stain and CD31 immunohistochemistry stain of the ankle are presented in Figure 3A. H&E stains revealed less severe synovium hyperplasia, inflammation and bone loss in CIA mice treated with dasatinib. Assessments of cartilage destruction using toluidine blue and safranin O stains revealed that the ankle joints from dasatinib-treated CIA mice exhibited marked alleviation of cartilage damage compared to ankle joints from vehicle-treated mice. CD31 immunohistochemistry was used to access pannus formation. Fewer vessels were observed in the dasatinib-treated group than the vehicle group. Vessels in the dasatinib-treated group were much smaller than the vehicle group. The histology scores of synovitis, inflammation, cartilage damage, bone erosion, and pannus formation in ankle joints from dasatinib-treated CIA mice were significantly lower than vehicle-treated CIA mice (Figure 3B). These results suggest that the administration of dasatinib inhibits the structural destruction of ankle joints in mice with CIA.

### Dasatinib Treatment Suppressed Inflammatory Responses in Mice With CIA

**Figure 4:**
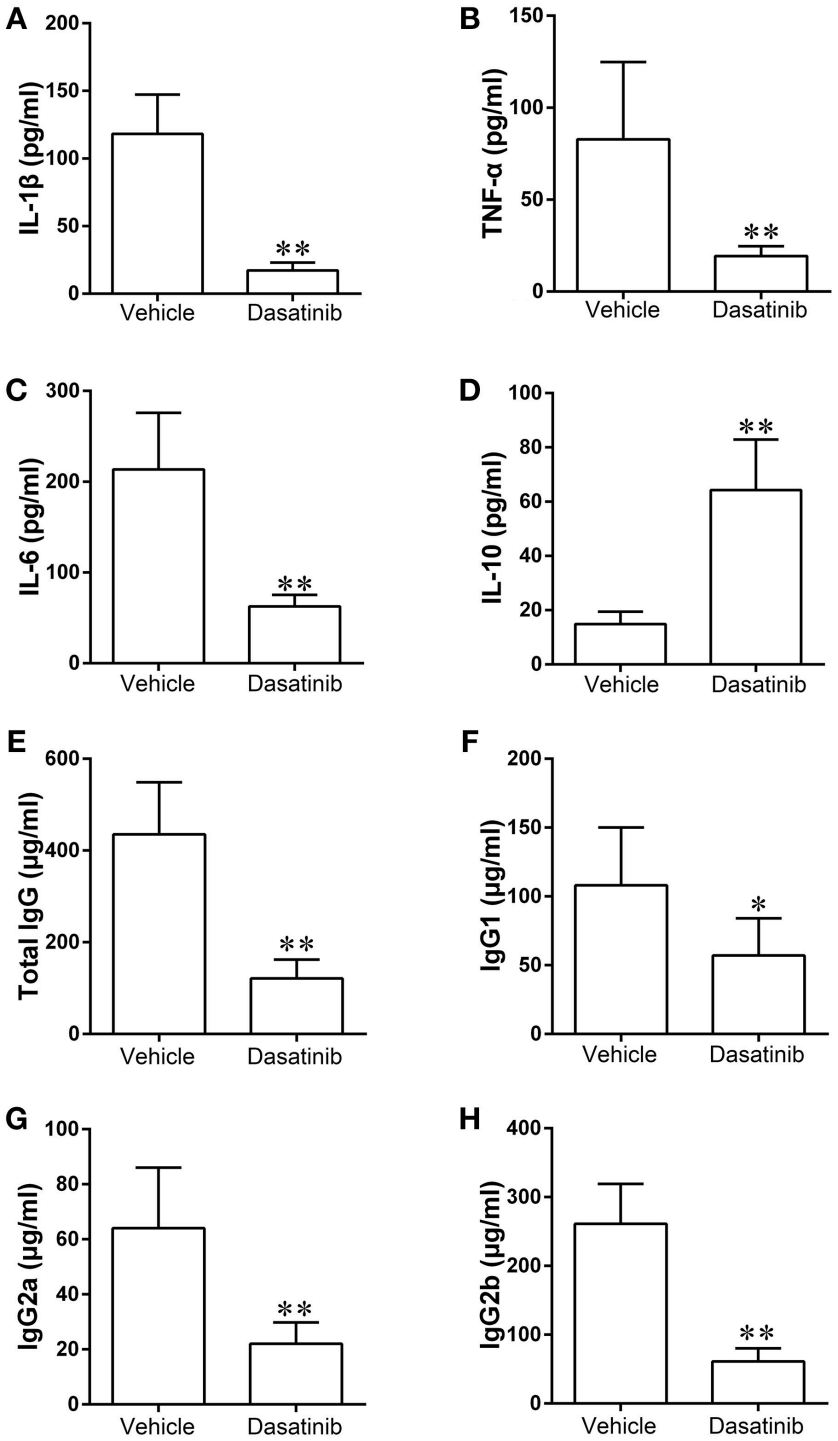
The levels of proinflammatory cytokines including IL-1β **(A)**, TNF-α **(B)**, IL-6 **(C)**, and anti-inflammatory cytokine IL-10 **(D)**, anti-mouse CII antibodies including total IgG **(E)**, IgG1 **(F)**, IgG2 **(G)**, and IgG2b **(H)** in serum samples of CIA mice were measured using ELISA. ^*^P < 0.05, ^**^P < 0.01 vs. vehicle group (*n* = 6 per group).

We measured the levels of proinflammatory cytokines including IL-1β, TNF-α, and IL-6, and the anti-inflammatory cytokine IL-10 in serum samples using ELISA to elucidate the mechanisms underlying the observed decrease in CIA severity following dasatinib treatment. The levels of IL-1β, TNF-α, and IL-6 were significantly lower in serum samples from CIA mice treated with dasatinib than vehicle-treated CIA mice (Figures 4A–C). The level of IL-10 in serum samples from the dasatinib group was markedly higher than the vehicle group (Figure 4D). These data suggest that dasatinib treatment exerts a therapeutic effect on CIA severity via inhibition of proinflammatory cytokines production, including IL-1β, TNF-α, and IL-6 and promotion of the anti-inflammatory cytokine IL-10.

### Dasatinib Treatment Decreased Serum Anti-mouse CII Antibodies in Mice With CIA

We determined the levels of total IgG, IgG1, IgG2, and IgG2b in serum samples to further investigate the mechanisms of dasatinib suppression of arthritis induction in mice. The expression of total IgG, IgG1, IgG2, and IgG2b decreased significantly in serum samples from CIA mice treated with dasatinib compared to vehicle-treated CIA mice (Figures 4E–H). These data indicate that the treatment effect of dasatinib in mice with CIA is associated with decreased serum level of anti-mouse CII antibodies.

### Dasatinib Suppressed the Migration of RA FLS

**Figure 5:**
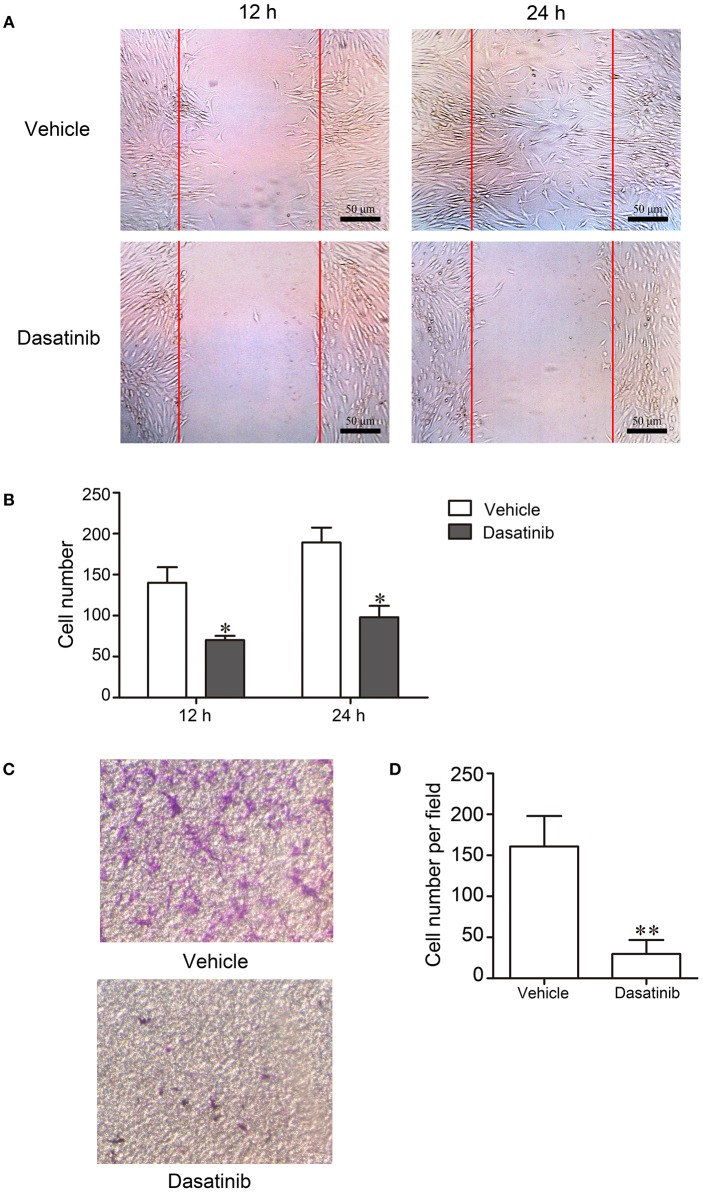
FLS from RA patients were cultured to investigate the mechanism of dasatinib treatment of CIA. Migration of cells was evaluated using the erasion trace test and transwell migration assay. **(A)** Microscope observation of FLS treated with dasatinib and control vehicles in the erasion trace test. **(B)** The number of cells that migrated to the wound region in 12 and 24 h. **(C)** Microscope observation of FLS that migrated through the porosity polycarbonate filters to the lower surface of the inserts. **(D)** The number of cells on the lower surface of the inserts in the transwell migration assay. ^*^P < 0.05, ^**^P < 0.01 vs. vehicle group (*n* = 3 per group).

We investigated the effects of dasatinib on FLS from RA patients to elucidate the mechanisms of dasatinib treatment on CIA. FLS were separated from synovial tissue of RA patients. The erasion trace test and transwell migration assay were used to assess cell migration. Cells treated with dasatinib migrated much slower than control cells treated with DMSO in the erasion trace test (Figure 5A). The number of cells that migrated to the wound region at 12 and 24 h was significantly lower in dasatinib-treated wells than control wells (Figure 5B). Only a few dasatinib-treated cells migrated through porosity polycarbonate filters to the lower surface of the inserts in the transwell migration assay (Figure 5C). The number of cells on the lower surface of the inserts in dasatinib-treated wells was much lower than the control wells (Figure 5D). These results indicate that dasatinib strongly inhibits the migration of FLS from RA patients.

### Dasatinib Inhibited RA FLS Proliferation

**Figure 6:**
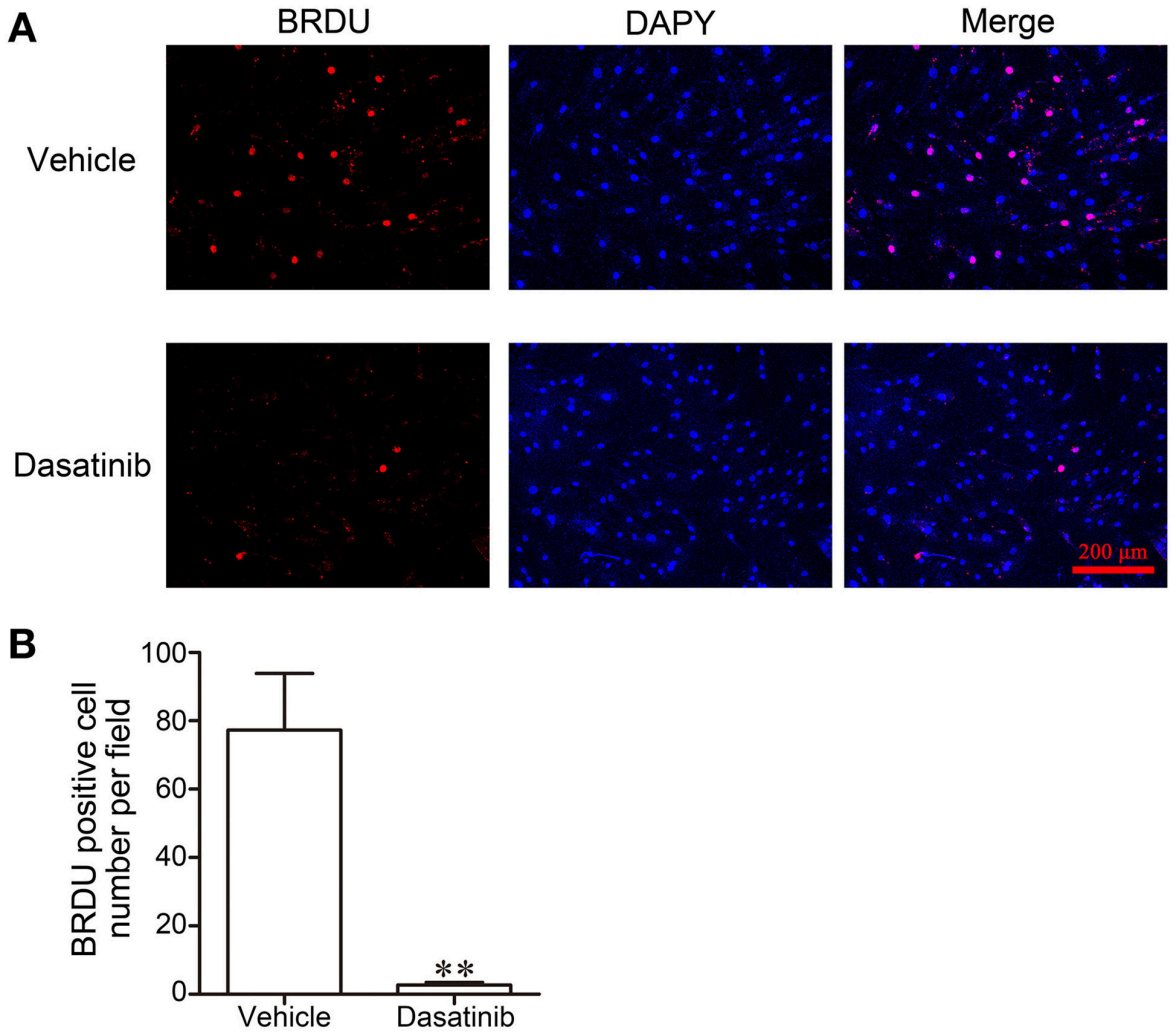
The effect of dasatinib on FLS proliferation in RA patients was evaluated using BrdU staining. **(A)** Microscope observation of FLS treated with dasatinib and control vehicle. **(B)** The number of BrdU-positive cells in each field. ^**^P < 0.01 vs. vehicle group (*n* = 4 per group).

5-Bromodeoxyuridine (BrdU) was used to evaluate cell proliferation. Cell proliferation was markedly inhibited after a 24 h treatment with dasatinib. Few BrdU-positive cells (TRITC, red) were observed in dasatinib-treated FLS (Figure 6A). The number of BrdU-positive cells was significantly larger in control FLS cells than dasatinib-treated cells (Figure 6B). This result reveals that dasatinib suppresses the proliferation of FLS from RA patients.

### Dasatinib Promoted RA FLS Apoptosis

**Figure 7:**
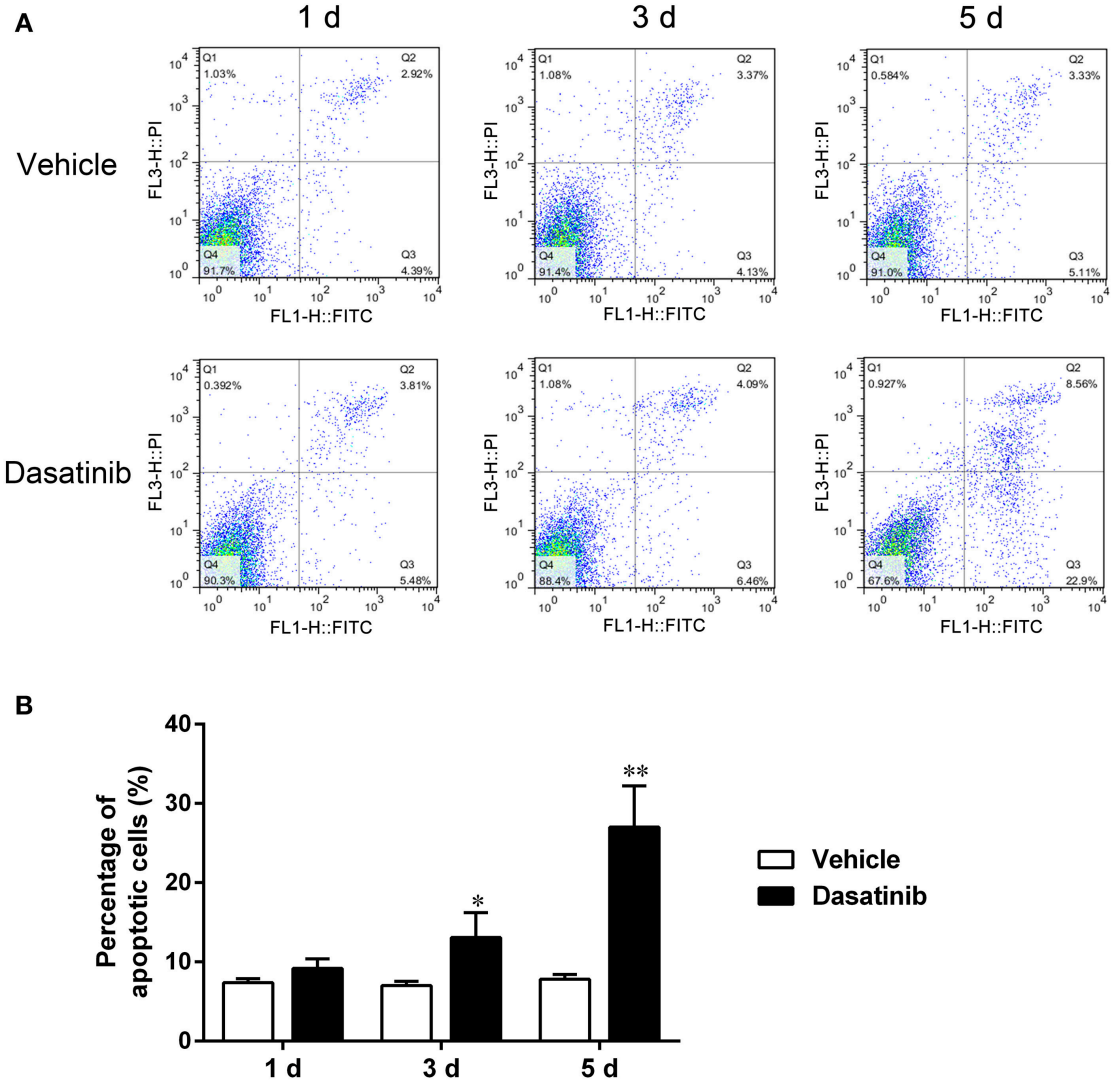
Flow cytometry was used to evaluate FLS apoptosis. **(A)** The percentages of early and late apoptosis cells treated with dasatinib and control vehicle for 0, 1, 3, and 5 d. **(B)** Quantitative analysis of the total percentage of apoptosis (including early and late apoptosis). ^*^P < 0.05, ^**^P < 0.01 vs. vehicle group (*n* = 3 per group).

Cell apoptosis was detected using flow cytometry. The percentages of early and late apoptosis increased after dasatinib treatment (Figure 7A). Quantitative analyses revealed that the total percentage of apoptosis (including early and late apoptosis) increased significantly after treatment with dasatinib for 3 and 5 d (Figure 7B).

### Dasatinib Down-Regulated mRNA Expression of MMP13, VEGF, FGF, and DKK1 in RA FLS

**Figure 8:**
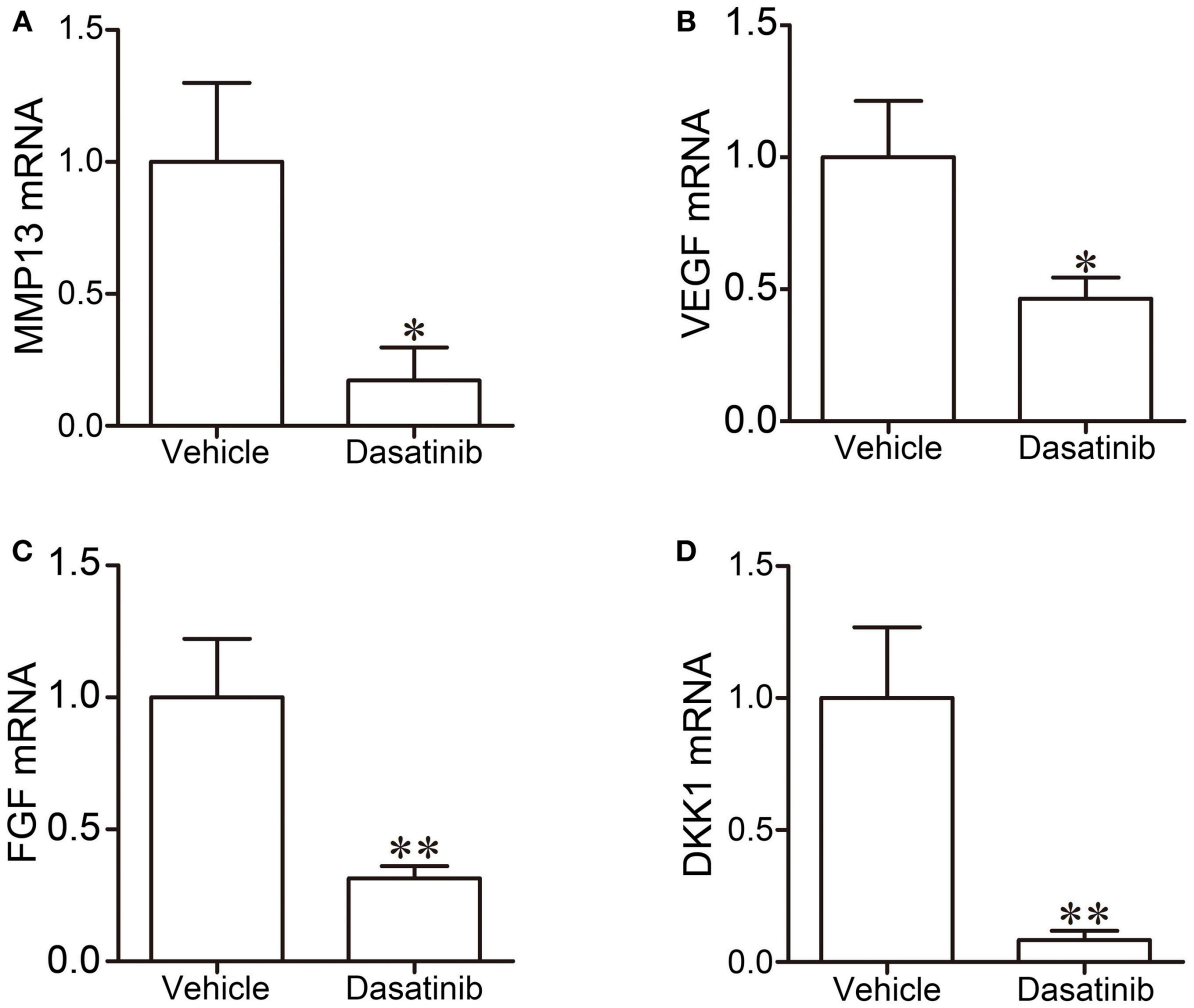
The mRNA expression of MMP13 **(A)**, VEGF **(B)**, FGF **(C)**, and DKK1 **(D)** in FLS was measured using RT-PCR. ^*^P < 0.05, ^**^P < 0.01 vs. vehicle group (*n* = 3 per group).

The mRNA expression of MMP13, VEGF, FGF, and DKK1 in FLS was measured using RT-PCR to further investigate the mechanism of action of dasatinib treatment on CIA. The mRNA expression of MMP13, VEGF, FGF, and DKK1 was significantly lower in FLS treated with dasatinib for 24 h than FLS treated with DMSO (Figure 8).

## Discussion

The present study investigated the treatment effect of dasatinib on CIA mice. The alleviation of arthritis symptoms and histopathological destruction was observed in CIA mice treated with dasatinib. We also found that dasatinib inhibited the migration and proliferation of FLS from RA patients, and dasatinib promoted RA FLS apoptosis. The mRNA expression of MMP13, VEGF, FGF, and DKK1 was down-regulated in FLS treated with dasatinib.

RA is a heterogeneous disease with a complex pathomechanism. Many cells participate in the pathophysiology of RA. The treatment of RA requires multi-target drugs. Dasatinib is a multitargeted inhibitor. Previous studies demonstrated that dasatinib exhibited strong inhibition of ABL and BCR/ABL tyrosine kinases and broad activity against Src-family tyrosine kinases (22–24). Dasatinib is a second-generation tyrosine kinase inhibitor. Tyrosine kinases play important roles in a variety of cellular events, such as differentiation, metabolism, proliferation, migration, and apoptosis (25, 26). Numerous tyrosine kinases participate in the pathophysiology of RA, including PDGFR, SCF-R/KIT, SRC, JAK, and SYK (27–29). These tyrosine kinases may be potential targets in RA therapy (30, 31).

Small-molecule tyrosine kinase inhibitors, such as imatinib, nilotinib, and dasatinib are used in the treatment of chronic myeloid leukemia. Imatinib exhibits preventive and treatment effects in CIA mice (11). Imatinib suppresses many signaling pathways implicated in RA pathological processes, such as mast cell c-Kit signaling, TNF-α release, macrophage c-Fms activation, cytokine production, and fibroblast PDGFR signaling and proliferation (11). Nilotinib exhibits different mechanisms than imatinib in the prevention of GPI-induced arthritis. Imatinib suppresses inflammatory and T-cell-derived cytokine production, and nilotinib only inhibits T-cell-derived cytokine production. Dasatinib exhibits similar targets and clinic use as imatinib and nilotinib (10), but it exhibits some differences in tyrosine kinase targeting and efficacy (13, 14). Dasatinib is more potent than the other two tyrosine kinase inhibitors (32).

Synovial tissue exhibits remarkable changes in RA. The synovium has two layers, an intimal lining layer and a sublining layer (3). Both layers undergo dramatic changes during RA pathophysiology. FLS, which are also known as type B synoviocytes, are an important component of the intimal lining layer. FLS are involved in many RA pathophysiological mechanisms. FLS play a vital role in cartilage degeneration, bone damage, inflammation reaction and pannus growth (4, 33–35). FLS increase in number and become more aggressive in RA. The balance between proliferation and apoptosis is disrupted. FLS exhibit strong resistance to apoptosis. FLS are key effector cells in RA and important target cells in RA treatment (3). The present study found that dasatinib inhibited the migration and proliferation of RA FLS and promoted RA FLS apoptosis. The amount of synovium also decreased in CIA mice treated with dasatinib.

Bone erosion is one striking features of RA. Osteoclasts are primarily responsible for bone destruction. Osteoclasts are activated in RA, and bone mass resorption occurs. Dasatinib demonstrated convergent bone anabolic and reduced bone resorption effects (36). It inhibited osteoclast formation in mouse primary bone marrow-derived monocytes and PC-3 cell-induced osteoclast differentiation (37). Dasatinib greatly alleviated bone erosion in joints and bone trabecular in mice with CIA. FLS play important roles in bone destruction. FLS produce massive amounts of inflammatory mediators and degradative enzymes in RA (38), including receptor activator of nuclear factor kappa B ligand (RANKL) (39, 40), which is essential for osteoclast differentiation and bone resorption (41, 42). FLS also prevent bone formation via osteoblast inhibition. The overexpression of Dickkopf-1 (DKK-1), which is an osteoblast inhibitor, was widely detected in RA synovium, especially in FLS (43). The expression of DKK-1 correlated with bone erosion and inflammation in RA (44). DKK-1 induces bone destruction and enhances the migration of FLS in RA (45, 46). Dasatinib suppressed the migration and proliferation of FLS from RA patients in the present study. The mRNA expression of DKK-1 was decreased in FLS treated with dasatinib. RANKL and DKK-1 expression in FLS may be downregulated because of the decrease in the number of FLS. We found that the mRNA expression of DKK1 was suppressed in FLS treated with dasatinib. This suppression may be an important reason for the alleviation of bone erosion by dasatinib in mice with CIA. These results indicate that the synovium is an important target in the treatment of RA with dasatinib.

Heightened inflammation always occurs in RA patients. The magnitude of the inflammatory response is a major cause of bone destruction and synovium invasion. The overexpression of proinflammatory cytokines, such as IL-1β, TNF-α, and IL-6, and low expression of the anti-inflammatory cytokine IL-10 are important factors in the RA pathomechanism (2). Anti-inflammatories are an important factor in RA therapy. Dasatinib exhibits anti-inflammatory effects. Dasatinib treatment suppressed the secretion of TNF-α in response to toll-like receptor (TLR) *in vitro* and *in vivo* (47). TNF-α levels in blood samples from a multiple sclerosis mouse model decreased significantly after dasatinib therapy (48). Dasatinib elevated the production of IL-10 and inhibited the expression of IL-6, IL-12p40, and TNF-α in TLR-stimulated macrophages (49). The present study found that dasatinib treatment alleviated paw redness and swelling. We also found that the levels of IL-1β, TNF-α, and IL-6 were significantly suppressed and that the level of IL-10 was markedly increased in serum samples of CIA mice treated with dasatinib. These results indicate that dasatinib suppresses inflammatory activity in mice with CIA.

Dasatinib exhibits potent DDR1 and DDR2 inhibitory activity (10). DDR2 participates in the pathological processes of RA (50). DDR2 is a key regulator of MMP13 expression (51), which is crucial to articular cartilage damage in RA. MMPs are potent destructors of the extracellular matrix. MMP13 exhibits high affinity for cartilage and bone degradation. MMP13 exhibits strong potency in the degradation of the cartilage proteoglycan aggrecan and collagen types I, II, and III, especially collagen type II. MMP13 expression was found in synovial tissues of RA patients but not healthy people (52). The levels of MMP13 mRNA are associated with the destructive course of RA (53). The expression of MMP13 mRNA was decreased in FLS treated with dasatinib. This effect may contribute to the DDR2 inhibitory activity of dasatinib. The inhibition of MMP13 mRNA may be an important mechanism of dasatinib treatment effect in CIA mice.

Angiogenesis plays a critical role in the pathogenesis of RA (54). The healthy two-layer lining structure becomes a pannus-like structure with hyperplasia of synovium in FLS of RA patients. More oxygen is consumed in the synovium because of the hyperplasia of FLS. This hypoxic environment stimulates vessel formation. Many newly formed blood vessels play an important function in the pathological changes of the synovium. FLS in RA are activated in a hypoxic environment and secrete growth factors, including VEGF and FGF (55, 56), which are key regulators of angiogenesis. The mRNA expression of VEGF and FGF were suppressed in FLS treated with dasatinib in the present study, and fewer pannus were observed in histopathological examinations of ankle joints of CIA mice treated with dasatinib. Previous study found that a host deficiency of DDR2 inhibited angiogenesis (57). These results suggest that dasatinib inhibits angiogenesis in RA synovium via suppression of DDR2.

In conclusion, we found that dasatinib exhibited a therapy effect in CIA mice. Dasatinib decreased the severity of inflammation and joint destruction. FLS are important target cells in dasatinib treatment of CIA mice. These results provide a promising and powerful strategy for RA treatment.

## Ethics Statement

All animal and human experiments were approved by the Ethics Committee of Xijing hospital, Fourth Military Medical University and were performed in accordance with institutional regulations. Synovium tissues were obtained from RA patients undergoing total knee replacement in the department of orthopedic, Xijing hospital. RA patients were informed and agreed to donate their synovium tissues for the experiment and signed written informed consent form before operation.

## Author Contributions

KG, JZ, XB, CY, QZ, and DZ conceived and designed the study. KG, JZ, XB, CY, XC, and HB performed the experiments. KG, XC, and HB wrote the paper. JZ, XB, QZ, and DZ reviewed and edited the manuscript. All authors read and approved the manuscript.

### Conflict of Interest Statement

The authors declare that the research was conducted in the absence of any commercial or financial relationships that could be construed as a potential conflict of interest.
